# *Saxifragaviridiflora* (Saxifragaceae), an unusual new species from Guangxi, China

**DOI:** 10.3897/phytokeys.184.73421

**Published:** 2021-10-25

**Authors:** Xin-Jian Zhang, Quan-Sheng Fu*, Jun-Tong Chen, Li-Juan Li, Peng-Rui Luo, Jing-Yi Peng, Xian-Han Huang, Hang Sun, Tao Deng

**Affiliations:** 1 CAS Key Laboratory for Plant Diversity and Biogeography of East Asia, Kunming Institute of Botany, Chinese Academy of Sciences, Kunming 650201, Yunnan, China; 2 University of Chinese Academy of Sciences, Beijing 100049, China; 3 CAS Key Laboratory of Plant Germplasm Enhancement and Specialty Agriculture, Wuhan Botanical Garden, Chinese Academy of Sciences, Wuhan 430074, Hubei, China; 4 College of Biology and Environmental Sciences, Jishou University, Jishou 416000, Hunan, China

**Keywords:** China, Guangxi, new species, Saxifragaceae, taxonomy

## Abstract

*Saxifragaviridiflora*, a remarkable new species of the genus Saxifragasect.Irregulares (Saxifragaceae) from Guangxi, is described and illustrated herein. This new species morphologically differs from all known S.sect.Irregulares taxa by its greenish petals, verruculose sepals, and thick leathery leaf blade abaxially scarlet with white spots.

## Introduction

*Saxifraga* Linnaeus, the largest genus of Saxifragaceae, comprises more than 440 species widely distributed throughout the Northern Hemisphere (Pan et al. 2001; Tkach et al. 2015a, b). Previous molecular phylogenetic studies suggested that *Saxifraga* is monophyletic, providing that S.sect.Micranthes (Haw.) D. Don is excluded (Soltis et al. 1996; Prieto et al. 2013; Deng et al. 2015; Tkach et al. 2015a, b). Recent molecular phylogenetic research covered at least 13 sections and 9 subsections within the genus (Tkach et al. 2015b). S.sect.Irregulares Haw., characterized by zygomorphic flowers with two elongated petals and stamens with club-shaped filaments (Tkach et al. 2015b), is the ancestral clade of *Saxifraga* first described by Haworth (Haworth 1803; Soltis et al. 2001; Zhang et al. 2015; Tkach et al. 2015b; Zhang et al. 2019b). This section currently comprises 16 species mainly distributed in East Asia (Magota et al. 2021).

China has a vast territory with a wide range of complex and diverse topographies and soils and covering several climate types, which contribute to the wealth of Chinese botanical diversity (Sun et al. 2017; Chen et al. 2018). Twelve species of Saxifragasect.Irregulares are native to China, including the recently reported new species, *S.daqiaoensis* F.G.Wang & F.W.Xing (Wang et al. 2008), *S.kegangii* D.G.Zhang, Y.Meng & M.H.Zhang (Zhang et al. 2017), *S.luoxiaoensis* W.B.Liao, L.Wang & X.J.Zhang (Zhang et al. 2018), *S.shennongii* L.Wang, W.B.Liao & J.J.Zhang (Zhang et al. 2019a), and *S.damingshanensis* W.B.Liao, W.Y.Zhao & J.H.Jin (Zhao et al. 2019).

In 2021, we inadvertently found a peculiar plant photograph posted on Baidu Tieba (https://tieba.baidu.com/), one of the most used Chinese communication platforms, and immediately deemed it to be a new species of Saxifragasect.Irregulares, as it possesses zygomorphic flowers with two elongated petals and stamens with club-shaped filaments, but its petals are greenish, which cannot be found in any existing species of Saxifragasect.Irregulares. We contacted the author of this photograph, Mr. Luo Dexuan, for phenological and geographical information regarding this specimen, and conducted fieldwork for this undescribed specimen. Subsequent morphological comparisons supported the status of the taxon as a new species, and it is described herein.

## Taxonomy treatment

### Saxifraga viridiflora

Taxon classificationPlantaeSaxifragalesSaxifragaceae

X.J.Zhang, T.Deng, J.T.Chen & H.Sun sp. nov.

AE4511AB-0F06-55A0-A079-ACAA5E5811E5

urn:lsid:ipni.org:names:77221295-1

Figs 1 , 2

#### Type.

China. Guangxi: Guilin City, Yongfu County, Baishou Town, 109°46'58.99"E, 25°5'15.5"N, 586 m alt., 27 June 2021, *X.J. Zhang*, *D.X. Luo Zhangxj98* (Holotype: KUN!; Isotypes: JIU!, SYS!).

#### Diagnosis.

*Saxifragaviridiflora* is easily distinguished from any other species of Saxifragasect.Irregulares by having greenish petals (vs. white petals). *S.viridiflora* morphologically resembles *S.epiphylla* and *S.kegangii*, but is distinct from the latter two in its leaf blade abaxially scarlet with white spots (vs. abaxially greenish/reddish with brown or yellow-green spots), and sepals with verruculose surface (vs. sepals without verruculose surface) (Table 1).

**Figure 1.**
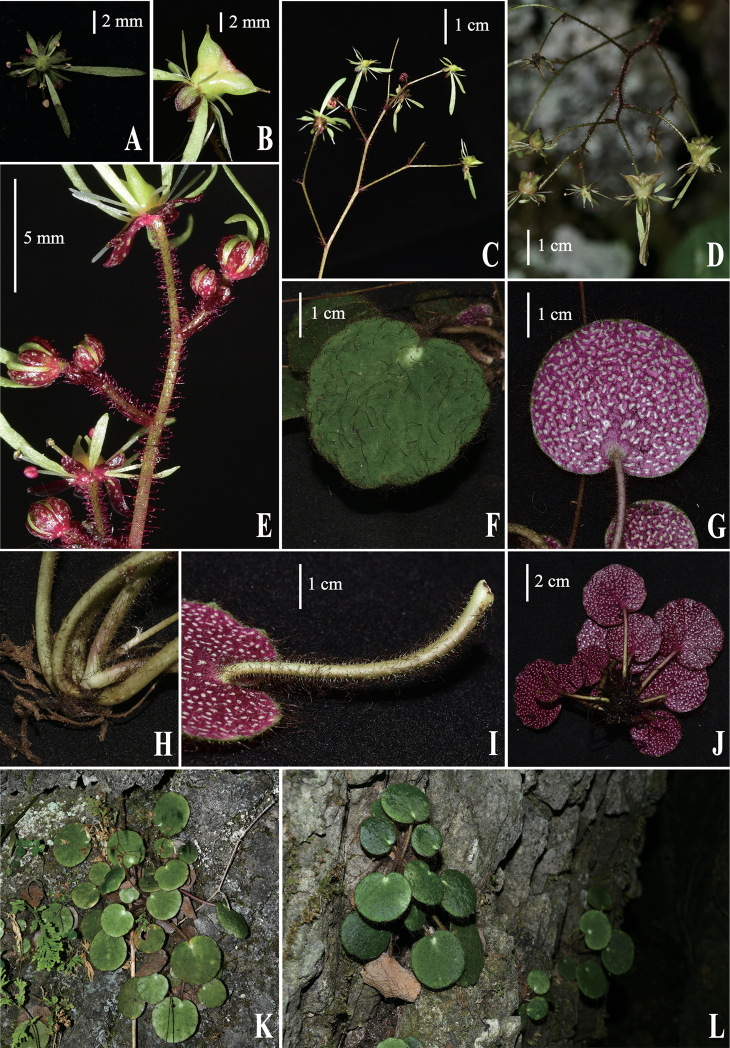
*Saxifragaviridiflora* X.J.Zhang, T.Deng, J.T.Chen & H.Sun, sp. nov. **A** flower, petals 5, greenish **B** fruit, capsule winged when mature **C** inflorescence **D** infructescence **E** pedicels glandular pubescent; sepals red, glabrous, abaxially white verruculose **F** adaxial leaf surface dark green, crisped villous **G, J** abaxial leaf surface scarlet, with white spotted, crisped villous **H** rhizomes crisped villous, petiole base unsheathed **I** petiole with crisped villous **K, L** plants and habitat.

**Figure 2.**
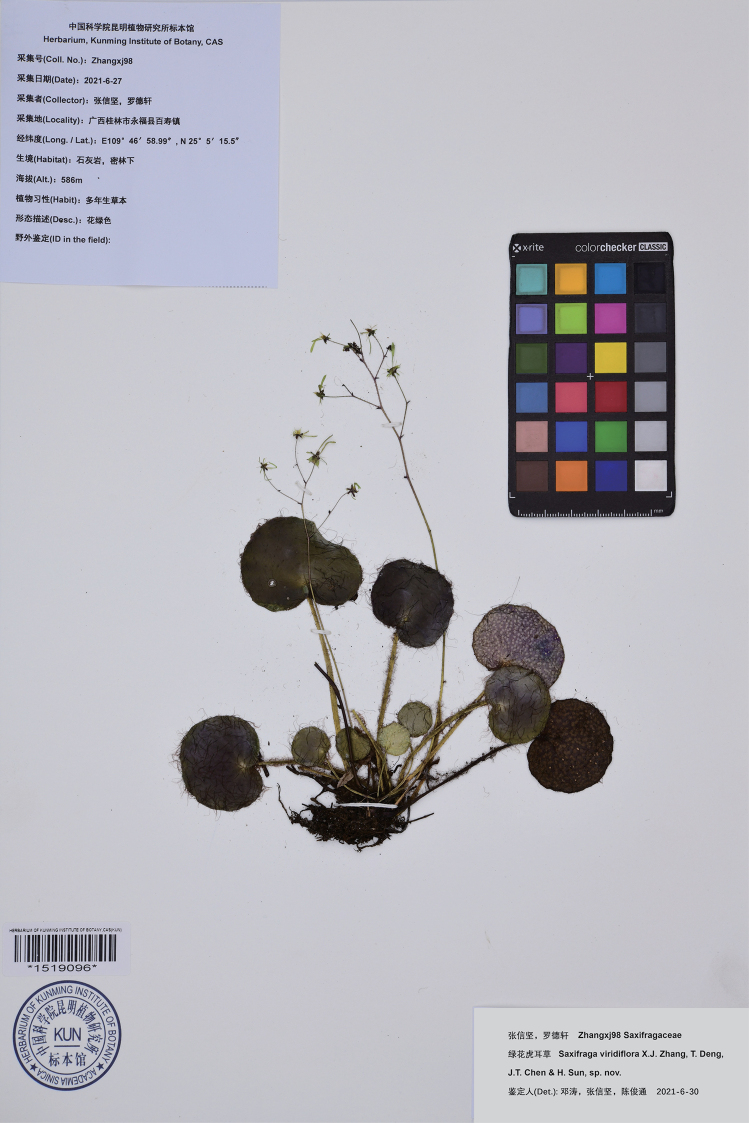
Photograph of the holotype of *Saxifragaviridiflora* X.J.Zhang, T.Deng, J.T.Chen & H.Sun, sp. nov. (Zhangx98, KUN1519096).

**Table 1.** Diagnostic characters of *Saxifragaviridiflora* and comparison with other related species of S.sect.Irregulares.

Characters	*S.viridiflora* sp. nov.	*S.epiphylla*	*S.kegangii*
Foliar embryo	absent	present	absent
Leaf shape	reniform to orbicular	ovate	fan-shaped to orbicular
Leaf margin	shallowly crenate to subentire	undulate, thickly dentate	entire or 8–10-crenate
Leaf texture	thick leathery to fleshy	leathery	leathery
Abaxial surface of leaf blade	scarlet, with white spotted	gray-green to red, with brown or yellow-green spotted	gray-green, with yellow-green spotted
Trichomes on leaf	both surfaces crisped villous	both surfaces glandular hispid	adaxially glabrous, abaxially glabrous or sparsely hispid
Petals	greenish	white, the base of three smallest petal with yellow spot	white, the base of three smallest petal with yellow spot
Sepals	red, glabrous, abaxially white verruculose	greenish, abaxially and marginally glandular hairy, without verruculose	greenish, abaxially and marginally glandular hairy, without verruculose

#### Description.

Perennial herbs, 12–30 cm tall. Stolons absent. Rhizomes rather short. Leaves all basal; petiole 5–12 cm long, crisped villous dark-purple (ca. 6 mm); leaf blade reniform, thick leathery, 2.5–4.0 cm long × 3.5–5.3 cm wide, base cordate, margin undulate, apex obtuse, both surfaces crisped villous dark-purple (5.0–9.0 mm long), adaxially greenish, abaxially purple or dark red, with white spots. Inflorescence paniculate, ca. 20 cm long. 5–10-flowered; branches 2.0–3.0 cm long, glandular pubescent, 1–2-flowered; pedicels slender, 1.0–2.0 cm long, glandular pubescent. Flowers zygomorphic; sepals 5, spreading to reflexed, narrowly ovate, 2.5–3.5 mm long × 1.5–2 mm wide, glabrous, abaxially red, with white verruculose, adaxially greenish, 3–5-veined, apex obtuse. Petals 5, greenish, margin entire, glabrous, apex acute; the three smallest lanceolate, 3.0–4.0 mm long × 1.0–1.2 mm wide, 3-veined; the two largest lanceolate oblong, 0.7–1.4 cm long × 1.0–1.2 mm wide, 3-veined. Stamens 10, 3.2–4.0 mm long. Ovary ovoid, 1.5–2.0 mm long, disc obscure; styles divergent ca. 1.0–1.8 mm long. Capsule beaks winged when mature, carpels 5–7 mm long × 3–4 mm wide. Seeds elliptic, the two sides slightly bent, ca. 0.6 mm long.

#### Etymology.

The specific epithet refers to the flowers of this new species that are green throughout the flowering period, differing from those of all other known Saxifragasect.Irregulares species. The Chinese name is given as “绿花虎耳草” (lǜ huā hǔ ěr cǎo), referring to the greenish petals of the new species.

#### Phenology.

In a two year personal observation of this new species in its native range, Guangxi. Guilin City (Luo Dexuan, pers. comm.), *S.viridiflora* was flowering from April to July and fruiting from June to August.

#### Distribution and ecology.

The new species, *Saxifragaviridiflora*, is currently known only from Yongfu County, Guangxi Province, China. It was observed to grow on dry limestone under dense jungles at altitudes between 500 and 600 m.

#### Paratypes.

China. Guangxi. Guilin City, Yongfu County, Baishou Town, 109°46'49.3"E, 25°5'16.1"N, 547 m alt., 10 July 2021, *X.J. Zhang*, *L.J. Li*, *J.Y. Peng*, *P.R. Luo Deng12030* (KUN); same locality, 575 m alt., 27 June 2021, *X.J. Zhang*, *D.X. Luo Zhangxj99* (KUN).

## Discussion

The new species *Saxifragaviridiflora* has zygomorphic flowers and stolons absent, which indicate a position in S.sect.Irregularesser.Rufescentes. *Saxifragaviridiflora* is distinct from all known sect.Irregulares taxa by its greenish petals, verruculose sepals, and thick leathery leaf blade abaxially scarlet with white spots.

Geographically, *Saxifragaviridiflora* was only found in Yongfu County of Guangxi Province, China. It grows only on dry rocks under dense jungles in limestone area, whereas other related species of sect.Irregulares usually grow on damp cliffs and rocks nearby valleys. Here we argue that the environmental heterogeneity plays an important role in the differentiation of the species in sect.Irregulares, given the leaf blade of *Saxifragaviridiflora* is thick leathery or fleshy (grow on dry rocks), while the leaf blade of other related species of sect.Irregulares are mainly papery or leathery (grow on damp rocks).

Notably, only seven species of Saxifragasect.Irregulares were recorded in “Flora of China” (Pan et al. 2001). However, six new species of S.sect.Irregulares were discovered in China in recent years, provided that *Saxifragaviridiflora* is counted. Furthermore, most of these new species were confined to a narrow geographical range. Species richness of S.sect.Irregulares has been quite underrated, and more field investigations and phylogenetic analyses are needed to infer its biodiversity and speciation history.

Since several new species of S.sect.Irregulares have been published in recent years, we include here an identification key to include all species known so far for this section.

### Identification key to Saxifragasect.Irregulares

**Table:** 

1	Stolons arising from axils of basal leaves, filiform	***S.stolonifera***
–	Stolons absent	**2**
2	Aerial stems developed; leaves cauligenous	***S.sendaica***
–	Aerial stems not developed; leaves radical	**3**
3	Leaf blade abaxially spotted	**4**
–	Leaf blade abaxially usually concolorous	**12**
4	Leaf blade elliptic to oblong, base cuneate	***S.kwangsiensis***
–	Leaf blade fan-shaped or ovate to broadly so, base cordate or peltate	**5**
5	Leaf blade with foliar embryos in sinus adaxially	***S.epiphylla***
–	Leaf blade without foliar embryos	**6**
6	Leaf base peltate	**7**
–	Leaf base cordate	**8**
7	Leaf blade papyraceous, apex acute	***S.mengtzeana***
–	Leaf blade thickly coriaceous, apex obtuse	***S.daqiaoensis***
8	Leaf margin lobed	**9**
–	Leaf margin shallowly crenate to subentire	**11**
9	Capsule beaks winged; leaf lobes margin irregularly dentate	***S.luoxiaoensis***
–	Capsule beaks divergent; leaf lobes margin entire	**10**
10	Abaxial surface of leaf blade purple spotted	***S.damingshanensis***
–	Abaxial surface of leaf blade yellow spotted	***S.shennongii***
11	Leaf blade abaxially gray-green with yellow-green spots	***S.kegangii***
–	Leaf blade abaxially scarlet with white spots	***S.viridiflora***
12	Longest petal serrate at margin	***S.fortunei***
–	Longest petal entire at margin	**13**
13	Leaf cleft	**14**
–	Leaf shallowly lobed	**15**
14	Upper petals nearly lanceolate, not spotted	***S.acerifolia***
–	Upper petals widely ovate, spotted	***S.cortusifolia***
15	Bracts leafy	***S.nipponica***
–	Bracts linear	**16**
16	Flowering stem and inflorescence reddish long glandular villous	***S.rufescens***
–	Flowering stem and inflorescence shortly glandular pubescent	***S.imparilis***

## Supplementary Material

XML Treatment for Saxifraga viridiflora
